# CAR T cells in pediatric systemic lupus erythematosus

**DOI:** 10.1007/s10067-025-07550-5

**Published:** 2025-07-11

**Authors:** Theodora Simopoulou, Athanasios Gkoutzourelas, Christina G. Katsiari

**Affiliations:** Department of Rheumatology and Clinical Immunology, Faculty of Medicine, School of Health Sciences, University of Thessaly, Larissa, 41 110 Greece

**Keywords:** B-cell depletion, CAR T cells, Pediatric lupus, Systemic lupus erythematosus

## Abstract

Pediatric systemic lupus erythematosus (SLE) is associated with high disease severity and significantly higher morbidity and mortality compared to adult-onset SLE, making it a particularly challenging condition to manage. Chimeric antigen receptor (CAR) T-cell therapy holds significant promise for improving outcomes in refractory pediatric SLE. However, the use of CAR T-cell therapy in pediatric patients presents unique challenges, including difficulty in collecting sufficient T cells. Pediatric patients may experience distinct side effects, such as age-specific toxicities or more pronounced neurotoxic events, requiring tailored monitoring and management. Additionally, the psychological impact on children and their families, facing potentially life-threatening treatment protocols, must be carefully addressed. To date, only four children with SLE have been treated with CAR T cells, highlighting the early stage of research in this area. The clinical outcomes provide hope that CAR T-cell therapy could offer a breakthrough in treating pediatric SLE; however, main limitations remain the small number of patients and the relatively short follow-up. More data are needed to fully assess the safety, efficacy, and long-term outcomes in this vulnerable population.

**Table:** 

Key Points • *Pediatric SLE is characterized by more severe manifestations, higher morbidity, and increased mortality compared to adult-onset SLE.* • *CAR T-cell therapy shows promise as a potential treatment for refractory pediatric SLE aiming ideally towards long-term, drug-free remission.* • *CAR T-cell therapy in pediatric SLE presents unique challenges, including age-related toxicities, technical difficulties, and ethical considerations.* • *To date, results are available from only a handful of children with SLE treated with CAR T cells. Expected advancements in CAR T-cell technology, along with data from ongoing clinical trials involving children with SLE, will determine the pace and extent of CAR T-cell therapy application in pediatric SLE.*

Key Points

• *Pediatric SLE is characterized by more severe manifestations, higher morbidity, and increased mortality compared to adult-onset SLE.*

• *CAR T-cell therapy shows promise as a potential treatment for refractory pediatric SLE aiming ideally towards long-term, drug-free remission.*

• *CAR T-cell therapy in pediatric SLE presents unique challenges, including age-related toxicities, technical difficulties, and ethical considerations.*

• *To date, results are available from only a handful of children with SLE treated with CAR T cells. Expected advancements in CAR T-cell technology, along with data from ongoing clinical trials involving children with SLE, will determine the pace and extent of CAR T-cell therapy application in pediatric SLE.*

## Introduction

Systemic lupus erythematosus (SLE) is a multifaceted autoimmune disease characterized by a wide spectrum of manifestations and involvement of multiple organs [1]. Although SLE primarily affects females of childbearing age, it can also be manifested during childhood, presenting unique challenges in diagnosis, management, and prognosis. Unlike adult-onset SLE (aSLE), childhood-onset SLE often involves more severe clinical manifestations and a higher risk of long-term organ damage, particularly affecting the kidneys, heart, and central nervous system [2].

The pathogenesis of SLE involves complex genetic, environmental, and immunological factors [3]. One of the hallmarks of the disease is the activation of autoreactive B cells [4]. Abnormal T-cell responses may further promote B-cell activation and production of autoantibodies, while aberrant clearance of apoptotic cells and subsequent formation of immune complexes contribute to organ damage [5].

Current therapeutic strategy includes glucocorticoids, immunosuppressants, and monoclonal antibodies aiming to suppress immune dysregulation. Given the central role of B cells in the pathogenesis of pediatric SLE, therapies targeting B cells have become a significant tool in everyday clinical practice [6]. Although this therapeutic strategy has shown efficacy, it fails to achieve drug-free remission.

Chimeric antigen receptor (CAR) T-cell therapy is a revolutionary approach that has transformed the treatment of B-cell hematological malignancies and is currently being explored as a potential therapeutic strategy for autoimmune diseases [7]. CAR T-cell therapy offers a more targeted approach as CAR T cells can be engineered to specifically target and eliminate the immune cells responsible for the autoimmune attack, such as autoreactive B cells or T cells. By selectively removing the harmful immune cells, CAR T-cell therapy may help restore a more balanced immune response, reducing inflammation and improving disease symptoms. Furthermore, the ability of CAR T cells to survive and proliferate within the host may be effective in treating refractory and relapsed autoimmune diseases, helping to achieve sustained long-term remission.

This review examines pediatric lupus, highlighting its distinct features and current treatment approaches, while also presenting the latest data on the emerging use of CAR T cells. We discuss the clinical experience derived from children with SLE who have been treated with CAR T cells, addressing key aspects such as potential side effects and the unique challenges associated with their use in the pediatric population.

### Pediatric SLE (pSLE)

Pediatric SLE (also known as juvenile SLE) refers to patients presenting SLE-related symptoms during childhood or adolescence. Currently, no precise age limit has been established to distinguish between patients with pSLE and patients with aSLE. In the majority of studies, pSLE refers to patients with SLE who are younger than 18 years old. About 15–20% of all SLE patients present with disease-related symptoms before the age of 18 [8]. The frequency of pSLE varies significantly, with an estimated incidence ranging from 0.36 to 2.5 per 100,000 children and a prevalence of 1.89 to 34.1 per 100,000. The average age of onset for pSLE is 12.6 years.

The diagnosis of pSLE is based on the SLICC and ACR/EULAR 2019 classification criteria [9, 10]. Currently, there are no specific criteria for SLE in the pediatric population. However, the similarities between pSLE and aSLE have led to the adoption and widespread use of these criteria for diagnosing pSLE as well.

In pSLE, similar to aSLE, pathophysiological mechanisms include impaired immune tolerance to self-antigens, genetic, epigenetic, and environmental factors. The uncontrolled production of autoantibodies activates dendritic cells, which produce type 1 interferons, promoting B lymphocyte differentiation into plasma cells, leading to the production of additional autoantibodies. B cells also secrete chemokines and cytokines, exacerbating the inflammatory response. The involvement of toll-like receptors in antibody-mediated activation of dendritic cells is significant. An imbalance between the formation and degradation of neutrophil extracellular traps further increases antibodies against nuclear antigens and impairs immune tolerance [3].

Clinical manifestations in pSLE may vary depending on age, severity, and organ involvement. pSLE may present with non-specific symptoms, such as fatigue, weight loss, headache, depression, memory or concentration problems, myalgia, arthralgia, and lymphadenopathy. More frequent disease-specific symptoms are mucocutaneous manifestations (rash, alopecia, Raynaud), fever, arthritis, renal, and neuropsychiatric involvement [11]. Lupus nephritis affects around 50–82% of children and may present with proteinuria, hypertension, and renal failure [12]. Neuropsychiatric involvement is encountered in a substantial number of patients (30–70%) and often appears early, within the first 2 years of disease onset. Cardiovascular manifestations, like pericarditis, occur in approximately 17–42% of pSLE patients, while ocular, gastrointestinal, and pulmonary manifestations have also been reported [13]. Hematologic manifestations include hemolytic anemia (9.3–38.6%) with a positive direct Coombs test, autoimmune thrombocytopenia (15–52.6%) and leucopenia, mainly lymphopenia (11.1–61.7%). Low complement levels and increased ANA are typical in pSLE patients. Serious complications include macrophage activation syndrome (MAS) leading to multisystem insufficiency and raised mortality rates [11]. Several clinical manifestations in pSLE patients account for increased disability rates in young patients and dictate the need for timely and effective multidisciplinary management to improve long-term outcomes [14].

### Pediatric vs. adult SLE

Pediatric and adult SLE are considered a continuum of the same disease. This is largely true for the majority of cases; however, there is growing understanding of the distinctive features of pSLE [15].

Sex distribution of pSLE patients depends on the age group of disease onset, so the female:male ratio is reported to be about 3.3:1 in pre-pubertal patients (< 7 years), 5.2:1 in peri-pubertal (7–13 years), 7.25:1 in post-pubertal (> 13 years), compared to the 9–10:1 ratio in adult onset SLE patients. The relatively lower female:male ratios reported among younger pSLE patients have been associated with genetic factors. In fact, the earlier the age of disease onset, the higher the frequency of monogenic lupus [16–18]. In addition, patients with an exceptionally early onset (before the age of 5) tend to present atypically, often lacking autoantibodies and experiencing more severe disease courses with poorer prognoses [19].

Bundhun et al. in their systematic review and meta-analysis reported that compared to aSLE, pSLE patients suffer more frequently from renal involvement and hematologic manifestations, seizure, ocular involvement, vasculitis, and fever (*P* < 0.0004). Conversely, pulmonary involvement, Raynaud phenomenon, photosensitivity, and malar rash were more frequently affecting aSLE patients (*P* ≤0.03) [20]. In the meta-analysis by Huang et al., however, pSLE patients suffer more frequently from malar rash, ulcers, or mucocutaneous involvement, as well as neurological and renal involvement, fever, cytopenias, lymphadenopathy, and cutaneous vasculitis in comparison to their adult counterparts. On the other hand, according to the same study, pSLE patients would be less frequently affected by articular manifestations or pulmonary involvement [21]. Methodological differences may account for some differences between studies.

Although no consensus exists on the differences between pSLE and aSLE across the full spectrum of organ involvement, several studies have shown the presence of increased damage at the time of diagnosis, and a higher prevalence of renal, neuropsychiatric, and cardiovascular involvement in patients with pSLE. Furthermore, pSLE seems to be more aggressive, with greater disease activity [19, 22–26]. Moe et al. assessed the mortality and survival rates of SLE patients in Norway from 1999 until 2022. The standardized mortality rate was substantially higher (7.2, 95% CI 3.3–14) in patients with pSLE [27]. Overall, pSLE patients require more aggressive treatments.

Treatment strategies in pSLE follow the paradigms established for aSLE. Only recently has a systematic comparison of treatment responses between pSLE and aSLE been attempted [28]. Due to safety and ethical concerns as well as inherent differences between pSLE and aSLE, new drugs approved for aSLE are also tested in clinical trials designed for pSLE, resulting, however, in a significant delay in the availability of novel treatment for children with SLE.

### Current treatment for pediatric SLE

SLE treatment currently relies on the use of corticosteroids along with conventional or biological immunosuppressants to control disease activity and prevent relapses.

#### Corticosteroids

Corticosteroids have traditionally been used in the treatment of pSLE, as first-line therapy both at initial diagnosis and for managing disease relapses [29]. Observational studies have implemented the improved outcomes and prevention of corticosteroid-related damage by the administration of repeated pulses of IV methylprednisolone and reduction of the orally administered prednisone in SLE patients [30–33]. pSLE patients are especially susceptible in presenting adverse events related to the corticosteroid administration. Therefore, it is essential to closely monitor blood glucose levels, arterial blood pressure, bone health, and growth (including weight, height, and pubertal development). Regular ophthalmological assessments are also crucial parameters for follow-up.

#### Antimalarials and conventional immunosuppressants

The vast range of adverse events following the long-term corticosteroid administration has led to the compelling need for steroid-sparing treatment. Antimalarial drugs are crucial for treating pSLE patients, serving as an effective steroid-sparing option. The Single Hub and Access point for Pediatric Rheumatology in Europe (SHARE) recommendations for the diagnosis and treatment of pSLE advise that all pSLE patients should regularly be prescribed hydroxychloroquine (HCQ) [29]. For children with pSLE, HCQ doses up to 6 mg/kg/day (based on lean body weight) are considered a safe treatment choice [29].

If the severity of the disease prevents tapering of oral prednisolone despite adequate adherence to oral prednisone and HCQ, a disease-modifying anti-rheumatic drug (DMARD) is the next therapeutic step to enhance disease control and allow for subsequent corticosteroid tapering. DMARDs usually used to treat pSLE patients include mycophenolate mofetil, azathioprine, methotrexate, or cyclophosphamide in severe cases [29]. Intravenous immunoglobulin (IVIG) and plasma exchange have also been used for severe pSLE cases.

#### Biologic DMARDs (bDMARDs)

The available data on the use, efficacy, and safety of biological agents in SLE treatment primarily come from studies involving patients with aSLE. Two biological agents have been mostly used in the treatment of patients with pSLE, rituximab and belimumab. Typically, indications for bDMARDs initiation in pSLE patients include lupus nephritis, mucocutaneous, musculoskeletal, hematologic manifestations, and difficulty in corticosteroid tapering. Other indications may include neurologic, pulmonary, vascular, and general manifestations refractory to the conventional treatment [34].

Rituximab (RTX) is a chimeric monoclonal antibody that binds to the cell surface protein CD20 of B cells, which is detected in both early pre-B cells and mature B cells, but not in differentiated plasma cells. Through this binding, it appears to induce antibody-dependent cellular cytotoxicity, complement-mediated cytotoxicity, and apoptosis of B cells [35]. The use of RTX in the treatment of pSLE has not received formal approval; however, it is practiced and supported by published data based on retrospective cohorts and case series, which reported more responders, improvement in disease activity (assessed using scores like SELENA-SLEDAI and BILAG), less frequent renal flares, and better prognosis in patients with renal and hematologic involvement. RTX treatment effectively lowered daily need for corticosteroids and maintained an acceptable safety profile. Adverse events associated with RTX administration in pSLE patients were mainly infusion-related reactions and infections [34].

Belimumab is a human monoclonal antibody against a B-cell activating factor (BAFF) (also known as B lymphocyte stimulator, BLyS). BAFF is responsible for the development and survival of B cells. In SLE patients, BAFF is overexpressed, leading to increased B-cell autoreactivity. Belimumab binds to circulating BAFF and inhibits its binding to the BAFF receptor on B cells, resulting in B-cell death. To date, there is only one randomized, placebo-controlled clinical trial assessing the safety and efficacy of belimumab in pSLE patients [36]. Belimumab received approval for pSLE patients aged 5–17 years in 2019 and for childhood onset lupus nephritis in 2022 [28, 36, 37]. Belimumab demonstrates a similar response profile in patients with aSLE and pSLE [36]. In general, belimumab, when used in combination with conventional DMARDs, is safe, clinically efficient, leads to a reduction of corticosteroid dose, and improves disease activity [34, 37].

Anifrolumab, a monoclonal antibody which binds to type I interferon receptor and inhibits the activity of type I interferons (interferon-α and interferon-β), is currently approved for the treatment of aSLE. Currently, there is an ongoing phase III, randomized, double-blind, parallel-group, placebo-controlled study (NCT05835310), to assess the pharmacokinetics, pharmacodynamics, safety, and efficacy of anifrolumab in pSLE patients [37].

More randomized clinical trials are needed in the pediatric population to better understand the safety and efficacy of bDMARDs in pSLE patients.

### Basic principles of CAR T-cell generation

**Fig. 1:**
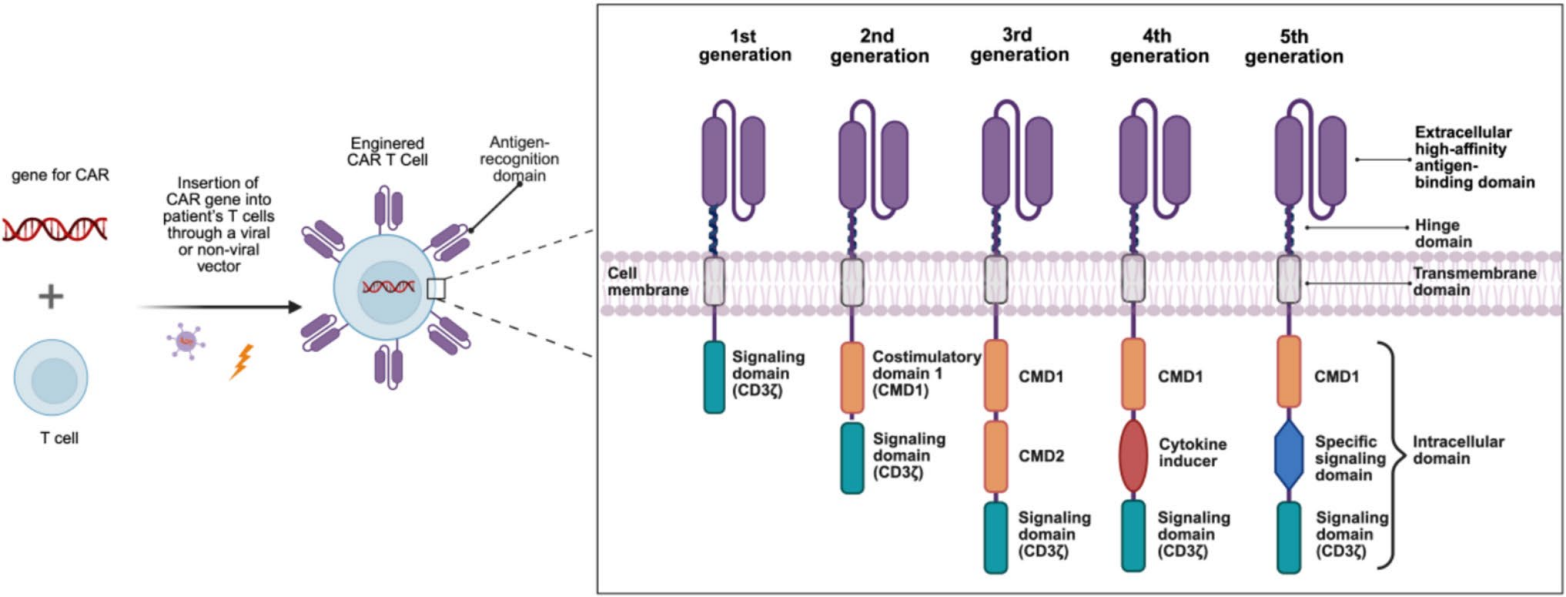
**CAR T-cell generations. **The CAR gene is introduced into T cells using various transfection methods, including both viral and non-viral approaches. CARs typically consist of four main components: an extracellular antigen-binding domain, a hinge domain, a transmembrane domain, and intracellular signaling domains. The first-generation CARs contain a CD3ζ signaling domain for signal transduction. The second-generation CARs incorporate an additional costimulatory domain, such as CD28 or 4-1BB, while third-generation CARs combine three intracellular domains (e.g., CD28 and 4-1BB). Fourth-generation CARs are based on the structure of the second-generation CARs, plus an activation element to induce cytokine production after antigen recognition. Fifth-generation CARs are engineered with an additional intracellular domain. CAR, chimeric antigen receptor “*Created in BioRender. Gkoutzourelas, T. (2025) *https://BioRender.com/8j7bi4k”

CARs are engineered synthetic receptors that typically consist of four main components: an extracellular high-affinity antigen-binding domain, a hinge domain, a transmembrane domain that provides anchorage to the plasma membrane, and intracellular signaling domains. Over the years, different CAR designs have been developed. Engineered CARs expressed in T cells are called CAR T cells. The number of signaling domains present in a CAR defines its generation (Fig. 1). The first-generation CAR T cells, which contained a CD3ζ or FcRγ signaling domain for signal transduction, were characterized by low in vivo persistence and are largely no longer in clinical use. The second-generation CAR T cells incorporate an extracellular antigen-recognizing domain, paired with two intracellular domains: CD3ζ and a costimulatory domain such as CD28-CD3ζ or 4-1BB-CD3ζ, while the third generation of CAR T cells combines three intracellular domains to exhibit different characteristics. These offer superior T-cell expansion and longer persistence. Fourth generation CARs are based on the structure of the second-generation CARs, plus an activation element to induce cytokine production after antigen recognition. Fifth generation CARs (also referred to as the next generation CARs) are engineered with an additional intracellular domain to activate some specific signaling pathways. CAR technology has now been extended also to CAR-Treg therapy [38]. Also, B-cell activating factor (BAFF) CAR T cells have been used in mouse models of SLE with promising results [39].

**Fig. 2:**
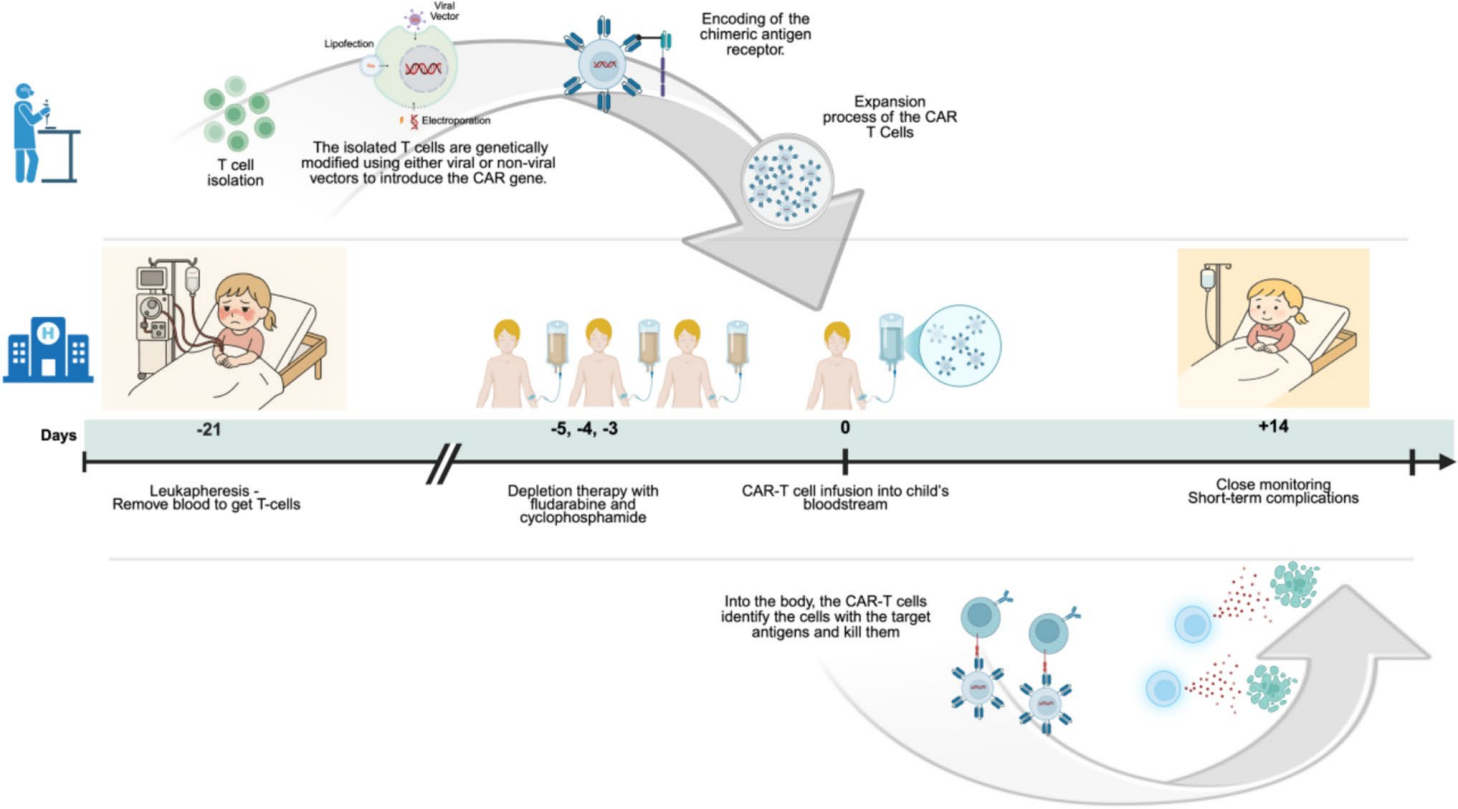
**Steps required for CAR T-cell therapy.** First, blood is removed to obtain white blood cells, including T cells, through a process called leukapheresis. Once isolated, T cells are sent to a specialized laboratory, where they are genetically modified to become CAR T cells and then expanded. Meanwhile, the child returns to the hospital to receive lymphodepleting therapy for three consecutive days. On the second day after completing lymphodepletion, the patient receives an intravenous infusion of CAR T cells. Close monitoring within the hospital for about 2 weeks is recommended. CAR, chimeric antigen receptor “*Created in BioRender. Gkoutzourelas, T. (2025) *https://BioRender.com/8j7bi4k”

The journey begins with leukapheresis, a process that extracts T cells from the patient’s blood. In a specialized laboratory, the isolated T cells are genetically modified using either viral or non-viral vectors to introduce the CAR gene. This gene encodes the chimeric antigen receptor, a unique protein engineered to recognize and bind to a specific target, an antigen on the surface of cells. The genetically modified CAR T cells are then subjected to an expansion process and finally infused back into the patient’s bloodstream [7]. Prior to CAR T-cell infusion, patients typically receive lymphocyte depletion therapy with fludarabine and cyclophosphamide in order to promote survival of infused CAR T cells [40]. Recognition of the particular antigen induces cytotoxicity and subsequent elimination of the targeted cells (Fig. 2).

CAR T cells are being engineered to combine an extracellular targeting function with specific intracellular domains that activate the cytotoxic potential of T cells. As they target CD19, a surface molecule that is present on most B cells through their development, they have the theoretical advantage of a wide spectrum of B-cell depletion. This pan-B cell approach seems appealing in disorders such as SLE in which numerous autoantigens are involved [41].

An argument for the use of CAR T cells in autoimmune diseases is that they show improved penetrance to peripheral tissues. Like other immune cells, CAR T cells keep the ability to migrate from bloodstream to tissues. Tue et al. showed that CD19 + and CD20 + B cells as well as follicular dendritic cells were depleted in the lymph nodes after CD19-CAR T-cell therapy, but not after RTX treatment. Additionally, non-lymphoid organs, including kidneys, were completely depleted of B cells [42]. As T cells can cross the blood–brain barrier, CAR T cells may also carry the dynamic of entering the central nervous system [43, 44].

An important aspect of CAR T-cell therapy is thought to be prolonged clinical remission. Patients who received CD19-targeted CAR T cells for B-cell malignancies have reached the milestone of a decade in remission [45]. Whether long-term remission might also be achieved in patients with autoimmune diseases is under study. In contrast to malignancies, where B-cell depletion may persist for years, CAR T cells in patients with autoimmune diseases tend to disappear, allowing B-cell reconstitution after a certain period [46]. Remarkably, despite the reappearance of B cells, autoantibodies remain undetectable and sustained drug-free remission has been documented [46, 47]. It is proposed that B-cell depletion by CAR T-cell therapy triggers an immune “reset,’’ ultimately leading to the restoration of the normal immune function. It remains an unanswered question whether continuous exposure to nuclear or intracellular antigens might trigger the production of autoreactive B cells again, as SLE is characterized by polyclonal B-cell activation.

### CAR T cells in pediatric SLE

In a pioneering clinical trial, autologous anti-CD19 CAR T cells were administered to a 20-year-old woman with severe treatment-refractory SLE, resulting in both serologic and clinical remissions [48]. A subsequent case series was reported with 15 patients, including eight patients with SLE aged 18–38 years old [49]. The study indicated that CAR-T therapy was well tolerated and induced rapid remission in patients. Notably, five of the eight SLE patients had onset of the disease before adulthood (at the age of 15 and 16 years). Thus far, more than 20 clinical trials of CAR-T therapy in patients with severe and refractory SLE are underway.

Clinical experience with CAR T-cell administration in children with SLE is even more limited (Table 1).

**Table 1:** Patients < 18 years old, treated with CAR T-cell therapy for SLE

Authors [ref]	Age (yrs)	Sex	DD (yrs)	Disease activity	Previous Tx	Protocol	Lymphodepletion	Outcome	Short-term toxicities	Follow up
Krickau et al. 2024 [50]	15	F	1	LN class IV (on dialysis) Rash Fever Arthritis SLEDAI-2 K = 23	HCQ AZA MMF BEL h–d GCs CYC	1 × 10^6^/kg of 2nd gen. CD19 CAR T cells Lentivirus vector	Fludarabine 12.5 mg/m^2^ on days − 5, − 4, and − 3 CYC 500 mg/m^2^ on day − 3	Nephritis resolved Arthritis resolved SLEDAI-2 K = 0	Transient neutropenia (grade 4) Malaise (days 3–7) CRS grade 1 Management: Antipyretics, TCZ 8 mg/kg on day 6 for CRS	6 mo
He et al. 2024 [52]	12	F	3	Rashes Ulcers Proteinuria Arthritis MAS SLEDAI-2 K = 12	GCs HCQ MMF BEL CsA	1 × 10^5^/kg of CAR T cells	Fludarabine 30 mg/m^2^ on days − 5, − 4, and − 3 CYC 300 mg/m^2^/day	No rash Ulcers healed Proteinuria resolved C3 normal (on day 28) Anti-dsDNA (−) (at 4 months) SLEDAI-2 K = 0	CRS grade 1 (mild fever on day 7) Management: ibuprofen ICANS grade 1 (tremors, dysgraphia, mild speech difficulties, mild lethargy on day 8) Management: 80 mg methyl-PNL IV, followed by 5 mg DEX/day IV × 3 days	5 mo
He et al. 2024 [52]	12	F	5	Hypertension Pleurisy LN class IV SLEDAI-2 K = 12	GCs HCQ CYC MMF RTX BEL TAC	1 × 10^5^/kg of CAR T cells	Fludarabine 30 mg/m^2^ on days − 5, − 4, and − 3 CYC 300 mg/m^2^/day	Pleurisy resolved Hematuria resolved Proteinuria: partial remission Anti-dsDNA (−) SLEDAI-2 K = 4	CRS grade 1 (mild fever on day 7) Management: ibuprofen	4 mo
Marasco et al. 2024 [51]	15	F	1	Hemolytic anemia Thrombopenia Malar rash LN class II/V Lungs: PAH Pericardial effusion SLEDAI-2 K = 22	HCQ GCs MMF CYC RTX	1 × 10^6^/kg of CAR T cells Lentivirus vector	Fludarabine 90 mg/m^2^ CYC 1500 mg/m^2^	Uranalysis normal PASP normal ProBNP normal SLEDAI-2 K = 2	Transient anemia (grade 2) Transient neutropenia (grade 3) CRS grade 1	10 wks

*AZA* azathioprine, *BEL* belimumab, *CRS* cytokine release syndrome, *CsA* cyclosporine-A, *CYC* cyclophosphamide, *DD* disease duration, *DEX* dexamethasone, *F* female, *GCs* glucocorticosteroids, *gen* generation, *HCQ* hydroxychloroquine, *h–d* high dose, *ICANS* immune cell associated autoimmune neurotoxicity syndrome, *LN* lupus nephritis, *MAS* macrophage activation syndrome, *Methyl-PNL* methylprednisolone, *mo* months, *MMF* mycophenolate mofetil, *NT-ProBNP* N-terminal pro-brain natriuretic peptide, *PAH* pulmonary arterial hypertension, *PASP* pulmonary artery systolic pressure, *RTX* rituximab, *SLEDAI* systemic lupus erythematosus activity index, *TAC* tacrolimus, *TCZ* tocilizumab, *Tx* treatments, *yrs* years, *wks* weeks

Krickau et al. reported successful use of a second-generation CD19 CAR T-cell therapy in a 15-year-old girl with rash, fever, and arthritis along with severe and rapidly progressive lupus nephritis [50]. Despite multiple medications, her condition worsened dramatically, and she required hemodialysis. After the administration of CAR T cells, SLE activity decreased promptly. Renal function improved, allowing for an extension of dialysis intervals and eventual cessation of dialysis on day 17. Mild cytokine release syndrome was managed with antipyretics and a single dose of tocilizumab. While the peak of CAR T-cell expansion was lower compared to adults, the cells persisted longer, remaining detectable 6 months post-infusion. Complete B-cell depletion was observed, and all autoantibodies, including anti-double stranded DNA (anti-dsDNA), disappeared [50].

A 15-year-old female patient in Italy, presenting with significant clinical activity—including hemolytic anemia, thrombocytopenia, nephritis, serositis, interstitial lung disease, and pulmonary hypertension—along with high titers of autoantibodies, was treated with anti-CD19 CAR T-cell therapy. After infusion, the patient showed clinical signs of mild cytokine release syndrome (fever, rash, and pleural effusion) and laboratory evidence of neutropenia and anemia. Clinical improvement was rapid, and complete peripheral B-cell depletion was achieved after 1 week [51].

Two 12-year-old female patients with refractory SLE were successfully managed with CAR T cells. The first suffered from relapses of skin rashes, ulcers, arthritis, and macrophage activation syndrome along with new onset of nephritis, while the other child experienced class IV lupus nephritis despite multiple immunosuppressant treatments. Disease duration was 3 and 5 years, respectively. They were treated with autologous CD19 CAR T cells, with significant clinical and laboratory improvement, while they achieved complete discontinuation of glucocorticoids and other immunosuppressants. Both patients developed mild cytokine release syndrome treated with antipyretics, while the first patient also experienced neurological symptoms indicative of immune effector cell associated encephalopathy (IECAE) managed with corticosteroids [52].

While the current literature provides valuable insights into CAR T-cell therapy in pSLE, several limitations must be acknowledged. The available data are limited to case reports involving only a small number of patients, aged 12–15 years old. Since adolescent patients tend to resemble those with aSLE, the efficacy of CAR T-cell treatments in younger patients remains unexplored. It is remarkable that all patients experienced a quick remission of the disease. However, drawing definitive conclusions is challenging due to the small sample size and variable follow-up periods. Moreover, the maximum reported follow-up was only 6 months. Data from aSLE patients include cases with sustained remission lasting up to 4 years. Whether similar long-term outcomes will be observed in pSLE patients remains to be determined. Ongoing clinical trials are investigating the safety and efficacy of CD19 CAR T cells in refractory SLE, both adult and pediatric, allowing inclusion of children aged ≥ 2 years (ClinicalTrials.gov ID NCT06585514, NCT06465147, and NCT06222853). A fundamental limitation of these studies is the lack of a control group. It remains ethically complex to establish a comparable patient cohort for such investigations. Overall, although the advent of CAR T-cell therapy has generated considerable excitement, more data on younger age groups, as well as on long-term safety and efficacy, are needed.

### Special considerations in children treated with CAR T cells

The development of CAR T-cell therapies for children has not progressed as rapidly as for adults. Currently, only tisagenlecleucel has received FDA approval for use in the pediatric population, showing efficacy in treating relapsed or refractory acute lymphoblastic leukemia [53].

Specific protocols regarding CAR T-cell therapy in children are lacking. The collection of autologous T cells using apheresis may be technically challenging, because patients often have low leukocyte counts. In pediatric leukapheresis, volume loss also requires continuing monitoring for anemia and electrolyte shifts. Collection procedures in pediatric patients typically last 3–4 h, which is generally longer than in adults. Additionally, it should be taken into account that the ability to collect sufficient cells by leukapheresis may be limited in small children and infants. Overall, the decision on eligibility should not be based on age, as stated in the 2021 best practice recommendation for the management of adults and children receiving CAR T-cell therapy [54]. Currently, a 3-week period is needed for the engineering and expansion process of CAR T cells. The child is admitted to the hospital to receive lymphodepleting chemotherapy (such as fludarabine and cyclophosphamide) usually 3–5 days before CAR T-cell infusion (Fig. 2) [55].

Close monitoring for complications after CAR T-cell infusion is recommended. The most prominent acute adverse events after CAR T-cell therapy are related to immune activation and subsequent cytokine release, particularly cytokine release syndrome (CRS) and immune effector cell–associated neurotoxicity syndrome (ICANS) [56]. CRS involves the excessive release of cytokines by immune cells. Typically, CRS clinically presents with fever as one of the earliest signs and may lead to life-threatening complications. Current therapeutic strategies to manage this immune-related adverse event combine symptomatic measures with corticosteroids and interleukin-6 receptor (IL-6R) blockade [57]. ICANS typically presents as toxic encephalopathy, with neurological symptoms such as decreased level of consciousness, headache, tremor, delirium, and seizures. These are observed within the first 2 weeks following infusion [56, 57]. Neurotoxic events associated with CAR T-cell therapy in pediatric patients have been a significant concern [58]. Studies indicate that approximately 40% of patients treated with CD19-targeted CAR T cells for B-cell acute lymphoblastic leukemia experience ICANS [59, 60]. The fact that children have an evolving central nervous system adds complexity to the use of CAR T-cell therapy in pediatric patients. The developing brain and nervous system in children may respond differently to neurotoxic events caused by CAR T cells compared to adults. This underscores the importance of monitoring and early intervention in pediatric population to help mitigate any potential long-term effects. The management involves early identification and grading of symptoms using established criteria [54]. Age-appropriate neurologic assessments must be used, with standard tools validated in young children. The use of Cornell Assessment of Pediatric Delirium (CAPD) in children is recommended twice daily [54, 61]. Treatment is individualized, depending on symptom severity. If corticosteroids are needed, dexamethasone is the first-line choice, as it crosses the blood–brain barrier. For isolated ICANS, tocilizumab is usually avoided because it could worsen symptoms, while anakinra appears to be a plausible candidate for refractory cases [62].

Cardiovascular complications, mainly hypotension, have also been reported in pediatric CAR T-cell studies [54].

The duration of post-infusion monitoring for children receiving CAR T-cell therapy varies and is based on clinical guidelines, institutional protocols, and patient-specific factors. While some protocols recommend a minimum of 2 weeks, extended monitoring periods may be necessary, particularly in the presence of complications.

So far, no cases of severe toxicity have been reported in children with SLE treated with CAR T cells (Table 1). Long-term monitoring regarding the risk of infections is crucial. Opportunistic infections are common after CAR T-cell therapy, and antimicrobial prophylaxis may be needed. B-cell aplasia and the resulting hypogammaglobulinemia are commonly present, and children may routinely need immunoglobulin replacement due to their immunological immaturity [53, 54, 60]. Further concerns arise due to the psychosocial aspects of pediatric CAR T-cell therapy, such as parental involvement and child-specific concerns, requiring supportive care both for patients and their caregivers [63]. Children and adolescents undergoing CAR T therapy often face prolonged hospitalizations, invasive procedures, and significant uncertainty regarding outcomes, all of which can lead to anxiety, fear, and depression. Furthermore, the intensive nature of therapy, with extended hospital stays and frequent outpatient visits, can disrupt schooling. For many children, the loss of routine and peer interaction during this critical stage of cognitive and social development can have both short-term and long-term impacts on their social life. Studies on survivors of childhood hematopoietic stem cell transplant exhibited poor sleep, fatigue, and anxiety that are known to impair cognitive domains such as memory and attention [64, 65].

The long-term effects of CAR-T therapy on a developing immune system and organ function remain uncertain, highlighting the need for ongoing vigilance regarding potential late-onset toxicities.

## Future perspectives

CAR T-cell treatment has the potential to revolutionize the treatment of autoimmune diseases, including pSLE. Future goals include better efficacy, sustainable results, minimal toxicity, and maximum accessibility.

To improve the ability of CAR T cells to reach and invade tissues, various engineering strategies are under development like armored CAR T cells, which are genetically modified to secrete cytokines (like IL-15) or other factors that can enhance their persistence in tissue and overcome immunosuppressive barriers [66, 67]. To balance efficacy with safety, switchable CAR T cells that can be activated on demand and deactivated once they have completed their task are under study [68].

Compared to traditional autologous CAR T cells, allogeneic universal CAR T cells could greatly expand the accessibility of such therapy to SLE patients. Key advantages of off-the-shelf allogeneic CAR T cells include reduced treatment costs, elimination of the need for leukapheresis and prolonged hospitalization, enhanced production scalability, and the use of T cells not affected by prior immunosuppressive therapies [69]. Currently, such an approach has been used in one adult patient with refractory myositis and two patients with diffuse systemic sclerosis [70]. A phase-I clinical study aiming to assess the safety and efficacy of the CD19 universal CAR T cells in the treatment of pediatric refractory SLE in children with age ≥ 5 years is underway (ClinicalTrials.gov ID NCT06691152.)

## Conclusion

CAR T-cell therapy holds significant promise for improving outcomes in refractory pediatric SLE. Unique challenges in this particular population dictate diligent monitoring and individualized care. To date, the main limitation remains the small number of patients and the relatively short follow-up.
